# Association Between Depression and Risk of Hypertension: A Systematic Review and Meta‐Analysis

**DOI:** 10.1002/brb3.70931

**Published:** 2025-09-25

**Authors:** Prakasini Satapathy, Abhay M. Gaidhane, Nasir Vadia, Soumya V. Menon, Kattela Chennakesavulu, Rajashree Panigrahi, Ganesh Bushi, Mahendra Singh, Sanjit Sah, Awakash Turkar, S. Govinda Rao, Khang Wen Goh, Muhammed Shabil, Edward Mawejje

**Affiliations:** ^1^ Center for Global Health Research, Saveetha Medical College and Hospital, Saveetha Institute of Medical and Technical Sciences Saveetha University Chennai India; ^2^ Jawaharlal Nehru Medical College, and Global Health Academy, School of Epidemiology and Public Health Datta Meghe Institute of Higher Education Wardha India; ^3^ Marwadi University Research Center, Department of Pharmaceutical Sciences, Faculty of Health Sciences Marwadi University Rajkot Gujarat India; ^4^ Department of Chemistry and Biochemistry School of Sciences JAIN (Deemed to Be University) Bangalore Karnataka India; ^5^ Department of Chemistry Sathyabama Institute of Science and Technology Chennai Tamil Nadu India; ^6^ Department of Microbiology IMS and SUM Hospital Siksha ‘O’ Anusandhan (Deemed to Be University) Bhubaneswar Odisha India; ^7^ School of Pharmaceutical Sciences Lovely Professional University Phagwara India; ^8^ Department of Biotechnology Graphic Era (Deemed to Be University) Dehradun India; ^9^ Graphic Era Hill University Dehradun India; ^10^ Department of Paediatrics Dr. D. Y. Patil Medical College Hospital and Research Centre Dr. D. Y. Patil Vidyapeeth (Deemed‐to‐be‐University) Pimpri Maharashtra India; ^11^ Centre Centre for Research Impact and Outcome Chitkara University Institute of Engineering and Technology Chitkara University Rajpura Punjab India; ^12^ Department of Data Science Gokaraju Rangaraju Institute of Engineering and Technology Bachupally Telangana India; ^13^ Center of Science and Information Technology International University Nilai Indonesia; ^14^ University Center for Research and Development Chandigarh University Mohali Punjab India; ^15^ School of Public Health Makerere University College of Health Sciences Mulago Hill Kampala Uganda

**Keywords:** cardiovascular risk, depression, hypertension, mental health, meta‐analysis, systematic review

## Abstract

**Background:**

Hypertension and depression are major global health concerns, with increasing evidence suggesting a potential bidirectional relationship between the two conditions. While previous studies have explored this association, the findings remain inconsistent, necessitating a comprehensive evaluation. This systematic review and meta‐analysis aimed to assess the association between depression and the risk of developing hypertension by synthesizing evidence from observational studies.

**Methods:**

A systematic search of PubMed, Embase, and Web of Science databases was conducted up to December 20, 2024. Observational studies that examined depression as an exposure and hypertension as an outcome were included. Random‐effects meta‐analysis was performed to estimate pooled odds ratios (ORs) and hazard ratios (HRs). Subgroup and sensitivity analyses were conducted to explore heterogeneity and assess the robustness of findings. Publication bias was evaluated using Egger test and funnel plots.

**Results:**

A total of 36 studies were included, comprising cross‐sectional, cohort, and case‐control designs. The pooled analysis indicated a significant positive association between depression and hypertension (pooled OR = 1.198, 95% CI: 1.086–1.321), with substantial heterogeneity (*I* ^2^ = 68%, *p* < 0.001). Longitudinal studies yielded a pooled HR of 1.277 (95% CI: 1.159–1.408) with no significant heterogeneity (*I* ^2^ = 0%). Subgroup analyses revealed stronger associations in cross‐sectional studies compared to cohort studies. Sensitivity analyses confirmed the robustness of findings, while publication bias was detected.

**Conclusion:**

This study provides evidence supporting a positive association between depression and an increased risk of hypertension. The findings underscore the importance of integrating mental health screening in cardiovascular care and adopting multidisciplinary strategies to address both conditions. Further research is needed to clarify causal pathways and explore targeted interventions.

## Graphical abstract

This meta‐analysis of 36 studies shows a significant link between depression and hypertension risk (OR = 1.198, 95% CI: 1‐086‐1.321; HR = 1.277, 95% CI: 1.159‐1.408). It advocates for mental health screening in cardiovascular care.

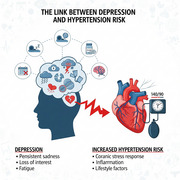

## 1 Introduction

Hypertension, a prevalent chronic condition characterized by sustained elevation of arterial blood pressure, poses significant health challenges globally. Treating hypertension can help reduce many of the fatalities caused by cardiovascular diseases (CVD), which are the world's leading cause of mortality (WHO 2023). It is a leading contributor to cardiovascular morbidity and mortality, affecting approximately 1.28 billion adults worldwide (Pokharel et al. 2022). While the physical health ramifications of hypertension, such as stroke, myocardial infarction, and kidney disease, are well‐documented (Bozkurt et al. 2016), its potential influence on mental health has garnered increasing attention. A contemporaneous review supported the findings of a positive association between comorbid anxiety and hypertension (Johnson 2019). In contrast to CVD, endothelial dysfunction, a precursor to atherosclerosis and atherothrombosis, is directly linked to inflammation. Additionally, endothelial dysfunction has been identified in depression and may serve as a phenotypic marker for this condition. Therefore, it will be crucial to comprehend vascular biology in relation to mental co‐morbidity (Halaris 2017). The intricate interplay between these two conditions has spurred a growing body of research, yet the precise nature and directionality of their relationship remain contentious and poorly understood.

Persistent sorrow, a lack of interest or enjoyment in activities, and a variety of physical and cognitive symptoms are the hallmarks of depression, which is one of the main causes of disability in the world (Lam 2018). According to the World Health Organization (WHO), > 280 million people suffer from depression globally, and its burden is projected to rise significantly in the coming decades (Organization WH 2019). Importantly, depression exerts profound systemic effects, including alterations in neuroendocrine, inflammatory, and cardiovascular pathways. Because of these systemic consequences, there is conjecture that depression and hypertension may have similar pathophysiological causes, including endothelial dysfunction, chronic inflammation, and dysregulation of the hypothalamic‐pituitary‐adrenal (HPA) axis (Gong and Deng 2023). Hypertension and depression frequently co‐occur, with evidence suggesting that individuals with hypertension may have an increased risk of developing depressive symptoms (Birk et al. 2019). Conversely, depression is a potential risk factor for the development and exacerbation of hypertension (Boukhari et al. 2024; Feng et al. 2024). This bidirectional association indicates the need for a nuanced understanding of the relationship between these two conditions. Such an understanding is vital for the development of integrated prevention and management strategies that address both the physical and mental health dimensions of affected individuals.

A substantial body of literature has explored the relationship between hypertension and depression, yielding mixed and sometimes contradictory findings. Observational studies, including cross‐sectional, longitudinal, and cohort designs, have reported varying degrees of association, influenced by factors such as study population, methodology, and confounding variables. Some studies suggest a positive association, where hypertension significantly increases the likelihood of developing depression (Khadoura et al. 2021), possibly mediated by factors such as medication side effects, lifestyle modifications, or the psychological burden of chronic illness. Other studies have found no significant association or even a potential protective effect of hypertension against depression in certain populations, a paradoxical finding that remains poorly explained (Graham et al. 2019).

The findings' variability might be due to variations in the operational definitions of depression and hypertension, research design, and demographic characteristics (such as age, sex, and ethnicity). Additionally, many studies have failed to account for important confounders, such as socioeconomic status (SES), comorbid conditions, and the use of antihypertensive medications (Camacho et al. 2018). Furthermore, the absence of standardized methods for measuring and diagnosing depression and hypertension across studies complicates comparisons and synthesis of findings. These discrepancies emphasize the necessity of a systematic review and meta‐analysis to thoroughly assess the available data and shed light on the relationship between depression and hypertension.

The relationship between hypertension and depression can be conceptualized through several theoretical frameworks. The stress hypothesis posits that chronic psychological stress, a key driver of depression, contributes to the development of hypertension via sustained activation of the HPA axis and sympathetic nervous system (Guilliams and Edwards 2010; Guo et al. 2024). Vascular remodeling, increased peripheral resistance, and eventually hypertension can result from elevated cortisol levels and elevated sympathetic tone (Hammer and Stewart 2006). Conversely, the vascular depression hypothesis suggests that cerebrovascular changes associated with hypertension, such as small vessel disease and reduced cerebral perfusion, may predispose individuals to depression by impairing neural circuits involved in mood regulation (Taylor et al. 2013). Additionally, the relationship between depression and hypertension may be complicated by common risk factors, such as drug abuse, poor eating habits, obesity, and a sedentary lifestyle (Bonnet et al. 2005; Du et al. 2023). The role of inflammation, a common denominator in both conditions, is also noteworthy. Pro‐inflammatory cytokines, including interleukin‐6 (IL‐6) and tumor necrosis factor‐alpha (TNF‐α), have been linked to depression and hypertension, suggesting a possible mechanism (Meng et al. 2020). Understanding these shared pathways is critical for disentangling the complex interplay between the two conditions. Another study highlighted the role of Ca^2+^/cAMP signaling, with its dysregulation linked to the pathophysiology of both depression and hypertension, establishing a clinical correlation between the two conditions (Bergantin 2020).

The present study aims to address these gaps by systematically reviewing the literature on the association between depression and the odds of developing hypertension and conducting a meta‐analysis to provide pooled estimates of effect sizes. By integrating findings from diverse studies, this analysis seeks to offer insights into the magnitude and directionality of the association, as well as the potential moderating effects of demographic, clinical, and methodological factors.

## 2 Methods

### 2.1 Study Design

The purpose of this study's systematic review and meta‐analysis is to assess the relationship between depression and the risk of hypertension. To maintain openness and rigor, the study was planned and presented in accordance with the Preferred Reporting Items for Systematic Reviews and Meta‐Analyses (PRISMA) criteria (Table S1). The International Prospective Register of Systematic Reviews (PROSPERO: CRD42024629409) has the protocol for the systematic review and meta‐analysis on file.

### 2.2 Literature Search

To find pertinent studies, a thorough search technique was used. We conducted a thorough search of Web of Science, PubMed, and Embase from the beginning until December 20, 2024. Keyword combinations and Medical Subject Headings (MeSH) associated with “depression” and “hypertension” were included in the search phrases. To find other acceptable research, we also looked through the reference lists of the included studies and pertinent review papers. There were no limitations on language or publishing date. The full search strategy for each database is provided in Table S2.

### 2.3 Eligibility Criteria

Studies were eligible for inclusion if they met several key criteria. The population of interest who had been diagnosed with depression, either through a clinical diagnosis or validated questionnaires. The existence of depression was the exposure of interest, whereas those without depression or with less severe depressive symptoms made up the comparative group. The primary outcome was the diagnosis of hypertension or elevated blood pressure, as defined by high blood pressure measured during an interview, prescription usage for antihypertensive drugs, or self‐reported or documented diagnosed hypertension that is greater than 140 mmHg (systolic) and/or 90 mmHg (diastolic). Only observational studies, including cohort, case‐control, or cross‐sectional designs, were considered eligible. This meta‐analysis only included studies that used the depression measure as a categorical variable; studies that used the depression measure as a continuous measure were not.

### 2.4 Data Extraction and Risk of Bias Assessment

To guarantee quality and consistency in data collection, two reviewers independently retrieved data from the chosen studies using a standardized form. Several important facets of each investigation were covered by the material that was retrieved. The study's features included study characteristics such as authors, publication year, study design, and setting; population demographics like sample size, age, and gender distribution; and definitions and measurement methods for depression and hypertension (Awad et al. 2024). Additionally, statistical data were retrieved, including any adjustments for possible confounders and effect estimates such as odds ratios (OR), relative risk (RR), or hazard ratios (HR), along with their corresponding 95% confidence intervals (CIs). Any differences throughout the data extraction process were discussed, and, if necessary, a third reviewer was engaged in order to establish a consensus. Data extraction were done using the Nested‐Knowledge software's “tagging” feature.

The Newcastle‐Ottawa Scale (NOS) was used by two reviewers to independently evaluate the methodological quality of the included research (Table S3) (Peterson et al. 2011). Each study was evaluated based on the following criteria: exposure and outcomes calculations, research group comparability, and participant selection.

### 2.5 Data Synthesis and Statistical Analysis

The diversity among the included studies was taken into account by doing a random‐effects meta‐analysis. Using a random‐effects model, the pooled OR and HR of hypertension in people with depression served as the main outcome measure. Statistical heterogeneity was assessed with the *I* ^2^ statistic (Shabil et al. 2024). Significant heterogeneity is indicated by *I* ^2^ values higher than 75%. R version 4.4 statistical software was used for all statistical studies (Alrahbeni et al. 2024; Shabil et al. 2023).

### 2.6 Subgroup and Sensitivity Analysis

To investigate possible causes of heterogeneity, such as research design (cohort, case‐control, and cross‐sectional), subgroup analyses were conducted. By eliminating one research at a time and tracking any changes in the overall results, sensitivity analyses were carried out using a leave‐one‐out strategy to assess the findings' consistency and robustness. In every analysis, *p*‐value of < 0.05 was considered statistically significant.

### 2.7 Publication Bias

To determine if publication bias existed, a funnel plot was made by plotting the effect measure against the inverse of its standard error (Swarup et al. 2024). Egger's test was used to determine how severe publication bias was.

## 3 RESULTS

### 3.1 Literature Search

**FIGURE 1:**
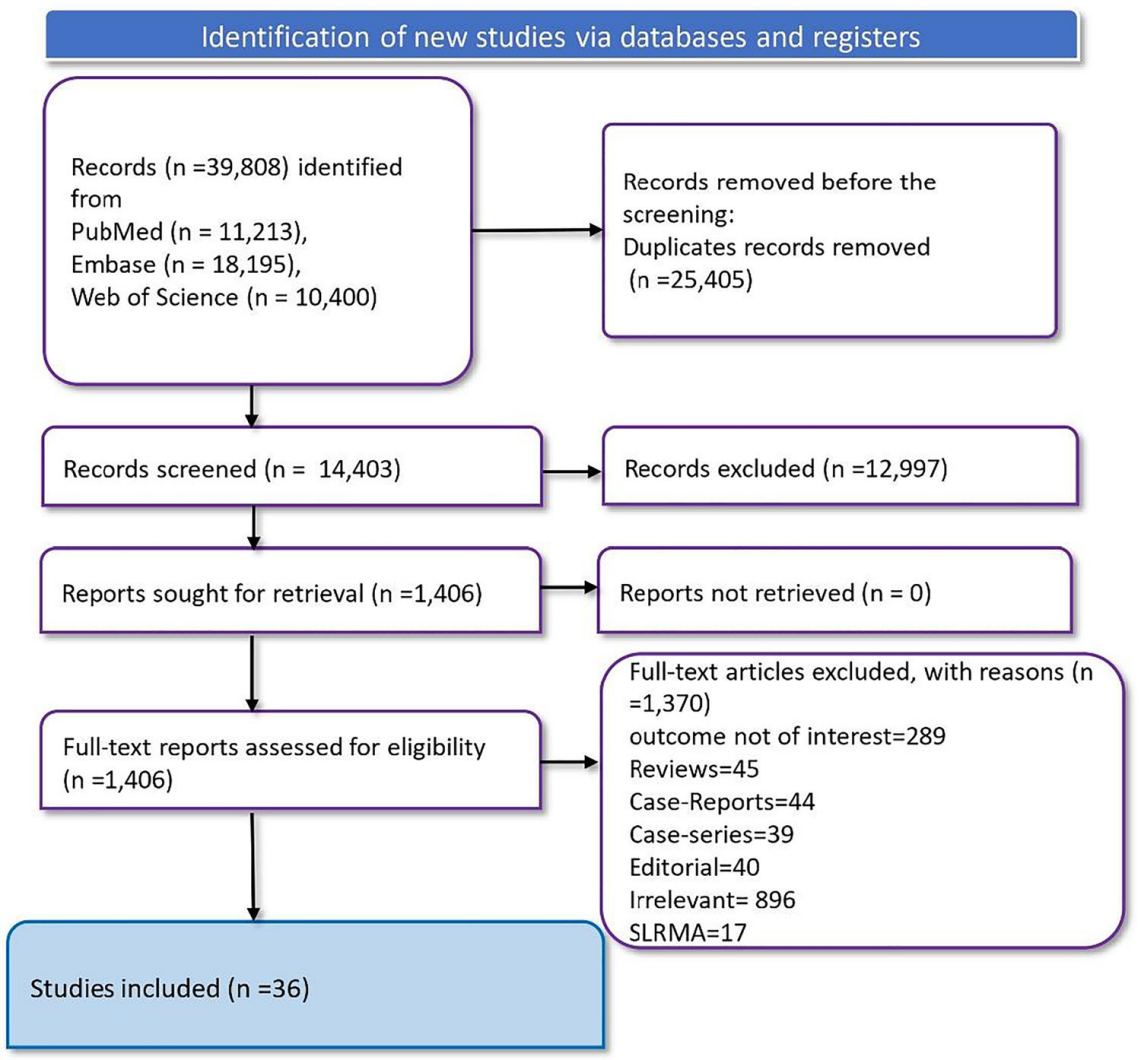
PRISMA flowchart depicting article selection and screening process.

This systematic literature search began with identifying 39,808 records across three major databases: PubMed (11,213 records), Embase (18,195 records), and Web of Science (10,400 records). After removing 25,405 duplicate records during the initial screening phase, 14,403 unique records remained for screening. Upon applying inclusion and exclusion criteria, 12,997 records were excluded, leaving 1406 reports that were sought for full‐text retrieval. Every one of the 1406 reports was successfully obtained and evaluated for eligibility. Of these, 1370 full‐text articles were excluded for various reasons: 289 had outcomes not of interest, 45 were reviews, 44 were case reports, 39 were case series, 40 were editorials, 896 were deemed irrelevant, and 17 were systematic literature review and meta‐analyses (SLRMA). Ultimately, 36 (Camacho et al. 2018; Ackerman‐Banks et al. 2023; Amaike et al. 2024; Blümel et al. 2020; Cai et al. 2022; Choong et al. 2023; Duman et al. 2024; Fernald et al. 2021; Flórez‐García et al. 2020; Gangwisch et al. 2010; Ginty et al. 2013; Grimsrud et al. 2009; Han et al. 2008; Jackson et al. 2016; Jonas et al. 1997; Kabir et al. 2006; Smith et al. 2024; Liu et al. 2024; Luo et al. 2023; Maatouk et al. 2016; Meyer et al. 2004; Munezero and Tomita 2021; Neyazi et al. 2024; Obas et al. 2022; Rhee et al. 2014; Schuchman et al. 2024; Shah et al. 2023; Stein et al. 2010; Tokioka et al. 2024; Wang et al. 2021; Wen et al. 2010; Wiehe et al. 2006; Yan et al. 2003; Yousuf et al. 2022; Zambrana et al. 2016; Zhao et al. 2023) Studies that satisfied all inclusion requirements were added to the final analysis (Figure 1).

### 3.2 Characteristics of Included Studies

Thirty‐six studies with diverse methodological approaches were included, comprising cross‐sectional studies, cohort/longitudinal studies, and a case‐control study. The studies spanned multiple continents, with the highest concentration in North America and Asia, followed by Europe, South America, Africa, and one study from Australia, representing a robust global perspective. Sample sizes varied substantially, ranging from 178 to 74,285,160 participants. The study populations predominantly consisted of adults (≥ 18 years), with some studies focusing on specific age groups such as middle‐aged and elderly populations (≥ 45 years) or young to middle‐aged adults (18–65 years). Gender distribution was reported in 24 studies, with male participation ranging from 0% to 56.7%, including five female‐only studies. For the longitudinal studies, follow‐up periods ranged from 3 to mean of 27.8 years. The Center for Epidemiologic Studies Depression Scale (CES‐D) was the most widely used depression evaluation tool. It was followed by the Hospital Anxiety and Depression Scale (HADS) and the Patient Health Questionnaire‐9 (PHQ‐9), while hypertension was primarily defined using standard blood pressure criteria of ≥ 140/90 mmHg or ≥ 130/80 mmHg, with some studies using self‐reported diagnosis, antihypertensive medication use, or a combination of criteria. Table 1 provides a thorough summary of the study's features.

**TABLE 1:** Characteristics of included studies.

Study	Country	Study design	Population characteristics	Sample size	Age (range/mean)	Male	Definition of depression	Definition of HTN	Follow‐up
(Ackerman‐Banks et al. 2023)	USA	Longitudinal	Pregnant individuals with deliveries during 2007 to 2019	119 422	≥ 15	0%	Mental Health Research Network (ICD‐ 9)/ICD‐ 10	ICD‐ 9/ICD‐ 10 code lists and added “not otherwise specified” CVD diagnosis codes based on review by Maternal Fetal Medicine physicians. New‐onset chronic HTN was defined from 43 days postpartum until 24 months postpartum because HTN within 42 days of delivery is considered a hypertensive disorder of pregnancy.	NA
(Amaike et al. 2024)	Nigeria	Cross‐sectional	Adult patients who are living with HTN	321	35–65	27.10%	The HADS was used to identify psychiatric morbidity (anxiety and depression)	Confirmed hypertensive patients (i.e., blood pressure above 140/90 mmHg	NA
(Blümel et al. 2020)	Chile	Cohort	All women who attended the service for preventive healthcare check‐ups, which is mandatory for public servants on an annual basis	1066	40–59	0%	Diagnosis is based on DSM‐IV criteria	Systolic/diastolic arterial blood pressure ≥ 140/90 mmHg and/or the use of anti‐hypertensive drugs	Mean = 27.8 years
(Cai et al. 2022)	USA	Cross‐sectional	Adults in NHANES	30,434	47.30 ± 0.24	48.98%	PHQ‐9, the total score of PHQ‐9 was 0–27 points, of which 0–9 was no depression, 10–14 was moderate depression, 15–19 was moderately severe depression, and 20–27 was severe depression. Study used 10 as the cut‐off for clinically relevant depression	Self‐reported and mean blood pressure value of three measurements ≥ 130 mmHg for systolic blood pressure or ≥ 80 mmHg for diastolic blood pressure	NA
(Camacho et al. 2018)	Chile	Cross‐Sectional	Chilean population included in the 2009–2010 National Survey of Health	4234	≥ 15	NA	Questionnaires	NA	NA
(Choong et al. 2023)	Malaysia	Cross‐sectional	Patients diagnosed with HTN with a duration of at least 6 months	1092	≥ 18	43.10%	Suspected depression when the total PHQ‐9 score was ≥ 10 points	HTN was defined as a systolic BP of ≥ 140 mmHg or a diastolic BP of ≥90 mmHg as diagnosed by a health care provider	NA
(Duman et al. 2024)	Turkey	Cross‐sectional	Patients aged ≥18 who attended the family medicine and cardiology outpatient clinic	199	56.4 ± 11	47.20%	HADS questionnaire, The scale classified patients as normal (0–7 points), borderline abnormal (8–10 points), or abnormal (11–21 points).	SBP ≥ 140 mmHg, DBP ≥ 90 mmHg, and/or the use of antihypertensive agents within 2 weeks.	NA
(Fernald et al. 2021)	Netherlands	Cohort	Adults from six ethnic backgrounds	22,165	18–70	41%	PHQ‐9 consists of nine items with a response scales 0 *not at all*, 1 ‘on several days’, 2 ‘on more than half of the days’ and 3 ‘nearly every day’	SBP ≥ 140 mmHg, or DBP ≥ 90 mmHg, or using BP lowering medication or asked to bring their prescribed medications to the research location	NA
(Flórez‐García et al. 2020)	Colombia	Cross‐sectional	Residents aged 27 years or older from the city of Medellín and its surrounding towns and villages	800	50.3 ± 12.1	45%	Depression was only assessed when perceived depression was detected according to DSM‐IV criteria (loss of interest in many things, such as hobbies or work activities that used to give them pleasure	Blood pressure was taken with the standard clinical procedure using an analogue blood pressure monitor, and the subjects were questioned about their use of medication to control their blood pressure.	NA
(Gangwisch et al. 2010)	USA	Longitudinal	Subjects for this study were participants in the 1982–1984, 1986, 1987, and 1992 follow‐up studies of the NHANES	4913	32–86	36%	To measure the presence of depressive symptoms, we used the standard cut‐off score of 16 out of a total possible score of 60 on the 20‐question CES‐D	SBP readings >140 mmHg or DBP readings >90 mmHg	10‐years
(Ginty et al. 2013)	UK	Cohort	Participants were selected from the Dutch Famine Birth Cohort, which comprises men and women who were born between November 1943 and February 1947.	455	HTN = 58.16 ± 0.88 Normotensive = 58.28 ± 0.94	47%	Possible caseness for both depression and anxiety, as measured using the HADS, is defined by scores of 8 or greater	SBP was measured twice in a row on two occasions (morning and afternoon) using an automated device	Mean = 5.5 ± 0.6 years
(Grimsrud et al. 2009)	South Africa	Cross‐sectional	Resident South Africans who lived in households and hostels during the field period of the study	4351	Mean = 37	46.30%	The World Mental Health pencil and paper (PAPI) version of the WHO Composite International Diagnostic Interview Version 3.0 (CIDI‐3.0) was used	HTN was assessed as self‐reported lifetime diagnosis in the section on chronic conditions in the questionnaire.	NA
(Han et al. 2008)	China	Case‐control	Participants older than 35 years, including both hypertensive patients and healthy controls, were randomly selected	NA	≥ 35	NA	NA	NA	NA
(Jackson et al. 2016)	Australia	Longitudinal	Women without a history of HTN at baseline	9182	49.5 ± 1.4	0%	Defined as CESD‐10 score ≥ 10, used of medication for depression/anti‐depressant use, or doctor‐diagnosed depression	Women were asked, “In the past 3 years have you been diagnosed with or treated for HTN?”	15‐years
(Jonas et al. 1997)	USA	Cohort	A population‐based sample of initially normotensive persons	2992	25–64	NA	NA	NA	7–16 years
(Kabir et al. 2006)	USA	Cross‐sectional	Participants from 561 families of the Bogalusa Heart Study	1017	12–62	48%	Symptoms of depression were obtained by self‐report using the CES‐D scale. A score of 16 or higher on the CES‐D scale was used as a screening tool to identify individuals with presumptive depression, a level where a clinical diagnosis of depression is likely	Participants were classified as having HTN if they reported taking antihypertensive medication or if they were < 20 years old and had a systolic and DBP > 95% of the BP values in their age group, or if they were greater than 20 years old and had an average SBP 140 mmHg or an average DBP 90 mmHg.	NA
(Smith et al. 2024)	USA	Cohort	FHS—Offspring Cohort Research Materials obtained from the NHLBI Biologic Specimen and Data Repository Information Coordinating Center	2390	Depression = 54.8 ± 8.1 No depression = 56.2 ± 8.0	48%	CES‐D scale was administered. Participants answered 20 items on a Likert‐type scale from 0 to 3, and responses were summed to produce a score from 0 to 60. We used a cut‐off point of ≥ 16 to identify “probable depression”	Measured by a doctor with a blood pressure cuff applied to a single arm while seated, and elevated blood pressure was defined as SBP ≥ 130 mmHg or DBP ≥ 85 mmHg at two consecutive readings	Median of 17.9 years (IQR = 14.6–18.8, range = 0.79–20.3)
(Liu et al. 2024)	China	Cross‐sectional	Participants aged 18 and above from the NHANES database	33,383	18–85	48%	PHQ‐9 screening tool, which is encompassed in the diagnostic criteria for depression outlined in the DSM‐IV	Mean SBP ≥ 130 mmHg or a DBP ≥ 80 mmHg or self‐reported diagnosis of HTN or self‐reported use of antihypertensive medication.	NA
(Luo et al. 2023)	China	Cohort	Middle‐aged and elderly population aged ≥ 45 years residing in local communities across China	11,050	45–80	48.50%	10‐item Scale (CES‐D‐10), categorized into: no depressive symptoms (ND) with scores < 10, depressive symptoms (DES) with scores between 10 and 14, and depression with scores above 14	SBP ≥ 140 mmHg and DBP ≥ 90 mmHg, self‐reported HTN diagnosed by a doctor, current use of antihypertensive drugs	5‐year
(Maatouk et al. 2016)	Germany	Cohort	Randomly chosen participants were visited at their homes by trained study doctors	3124	57–84	NA	PHQ‐8 was used to assess depression symptom. The total sum score of all items of the PHQ‐8 ranges between 0 and 24 points. A cut‐off point of at least 10 allow sone to differentiate ‘moderate‐to‐severe symptoms’ (Boukhari et al. 2024; Feng et al. 2024; Khadoura et al. 2021; Graham et al. 2019; Camacho et al. 2018; Guilliams and Edwards 2010; Guo et al. 2024; Hammer and Stewart 2006; Taylor et al. 2013; Bonnet et al. 2005; Du et al. 2023; Meng et al. 2020; Bergantin 2020; Awad et al. 2024; Peterson et al. 2011) from ‘no presence of, or mild depressive symptoms’ (0–9; referent)	SBP > 140 mmHg or DBP > 90 mmHg was classified as hypertensive or current use of antihypertensive drugs.	8‐year
(Meyer et al. 2004)	USA	Cohort	The Epidemiologic Catchment Area Study is a community‐based study of the prevalence of mental disorders supported by the NIMH	1049	≥ 18	40%	Psychiatric diagnosis in each site by using the Diagnostic Interview Schedule (DIS) based on the Diagnostic and Statistical Manual of Mental Disorders (DSM‐III)	Questions regarding HTN: (Organization WH 2023) Have you ever had high blood pressure? (Pokharel et al. 2022) Do you have high blood pressure now? and (Bozkurt et al. 2016) Are you receiving regular care from a health professional such as a doctor or nurse practitioner for this condition? Incident cases were defined as those with self‐reported HTN in 1993 who reported never having HTN in 1981 and 1982 (Waves I and II).	12‐year
(Munezero and Tomita 2021)	South Africa	Cross‐sectional	Female African Refugees in Durban	178	32 ± 11	0%	20‐item CES‐D scale, total score ≥ 24 was considered severe depressive symptoms	Individual with BP greater than 130/90 mmHg and above	NA
(Neyazi et al. 2024)	Afghanistan	Cross‐Sectional	Patients who were admitted to hospitals and underwent comprehensive interviews	2059	5–110	46%	19‐item Dari version of the CES‐D Scale: a score of 16 or higher indicated the presence of depression	Participants exhibiting a systolic blood pressure equal to or exceeding 140 mmHg, a diastolic blood pressure equal to or exceeding 90 mmHg, or meeting both criteria were classified as individuals manifesting hypertension	NA
(Obas et al. 2022)	Switzerland	Cohort	Participants with complete data on systolic and diastolic BP at first had complete baseline data on depression, potential confounders, and mediators; did not report a physician diagnosis of HTN or CVD at baseline; had no history of antihypertensive treatment at baseline; and were normotensive	3214	18–60	47.50%	Self‐reported, physician diagnosed depression or a history of antidepressant use or an SF‐36 MH score < 50	If they had physician‐diagnosed HTN or used antihypertensive medication, regardless of the BP level at corresponding health examination	10‐years
(Rhee et al. 2014)	Korea	Cross‐sectional	Patients who underwent routine health examinations and provided complete data on depression	30107	Depressive symptoms = 47.5 ± 10.7 No Depressive symptoms = 47.1 ± 10.4	NA	Severity of subjective depressive symptoms was assessed using the BDI, a 21‐item self‐report questionnaire that scores each question from 0 to 3 points (total score = 0–63 points). Patients with a total BDI score ≥ 16 were classified as having clinical depressive symptoms	SBP ≥ 130 mmHg or DBP ≥ 85 mmHg or use of antihypertensive medication	NA
(Schuchman et al. 2024)	USA	Cohort	Adults and children enrolled in the Nephrotic Syndrome Study Network (NEPTUNE)	745	9–50	NA	PROMIS scores were developed to improve and standardize patient‐reported outcomes to assess HRQOL	Hypertensive BP was defined as an SBP ≥ 130 mmHg or DBP ≥ 80 mmHg for adults and children ≥ 13 years of age	Adults = 36 months (IQR 18–53), Child = 35 months (IQR 19–49)
(Shah et al. 2023)	USA	Cross‐sectional	Low‐income participants who completed HTN and disability questionnaires	7,42,85,160	≥ 18	44.40%	Self‐reported frequency of depression‐related feelings and self‐reported use of depression medication	Questionnaire	NA
(Stein et al. 2010)	Colombia, Mexico, USA, Belgium, France, Germany, Italy, Netherlands, Spain, and Japan	Cross‐sectional	Participants in the WHO World Mental Health (WHM) Surveys	18,600	21–98	47%	All surveys used the WMH Survey version of the WHO Composite International Diagnostic Interview (WMH‐CIDI, now CIDI 3.0). Disorders were assessed using the definitions and criteria of the DSM‐IV	Respondents were asked, “Did a doctor or other health professional ever tell you that you had any of the following illnesses…HTN?” Respondents were also asked how old they were when the condition began. Only those reporting HTN onset after the age of 20 were included in the current analyses	NA
(Tokioka et al. 2024)	Japan	Cross‐sectional	Participants with normotension measured at research center	6705	55.7 ± 13.7	25%	CES‐D self‐reporting questionnaire was used for assessing depression. A cut‐off score of 15/16 is widely applied to screen for depression in Japan	SBP ≥ 140 mmHg and/or DBP ≥ 90 mmHg using BP measured at the research center	NA
(Wang et al. 2021)	China	Cross‐sectional	Hypertensive subjects in primary care setting	1856	53.7 ± 12.5	56.70%	Depression was identified as a score of ≥ 8 in the HADS‐D	SBP ≥ 140 mmHg, and/or DBP ≥ 90 mmHg, and/or use of antihypertensive agents within 2 weeks	NA
(Wen et al. 2010)	China	Cross‐sectional	Chinese nonagenarians and centenarians	687	93.51 ± 3.35	33%	Depression symptom was measured with GDS‐CD test. GDS‐CD includes 23 items (each has two options: yes, or no), which index depression in the old. GDS‐CD is commonly used for depression test in old people in Chinese, and a score above 10 on it usually defined as depression	HTN was defined as an SBP >140 mmHg and/or a DBP >90 mmHg and/or receiving anti‐hypertensive treatment	NA
(Wiehe et al. 2006)	Brazil	Cross‐sectional	Participants living in the urban area of Porto Alegre, a state capital with > 1.5 million inhabitants	1174	44.77 ± 17.2	44%	Major depression was diagnosed using the criteria from the fourth edition of DSM‐IV	HTN was diagnosed by SBP >140 mmHg or DBP >90 mmHg (mean of two measurements) or use of blood pressure‐lowering agents. For this analysis, blood pressure was classified according to the Seventh Report of the JNC on Prevention, Detection, Evaluation, and Treatment of High Blood Pressure (JNC VII) criteria	NA
(Yan et al. 2003)	USA	Longitudinal	Black and white adults from 4 US metropolitan areas	3308	18–30	NA	CES‐D scale with 20 items and 4 response choices for each item, scores ranging from 0 to 60	HTN was defined as an SBP >140 mmHg and/or a DBP >90 mmHg and/or receiving antihypertensive treatment	15‐year
(Yousuf et al. 2022)	Pakistan	Cross‐sectional	Adult patients admitted with uncontrolled HTN	290	≥ 18	50.30%	HADS‐D categorized as >8 and <8. A score of >8 on either scale was suggestive of having depression	SBP of >140 and a DBP of >90	NA
(Zambrana et al. 2016)	USA	Cohort	Hispanic women at baseline depressive symptoms and past depression history were measured	4680	50–79	0%	Psychosocial questionnaire using 6‐items from the CES‐D scale to assess depressive symptoms. CES‐D and categorized a score of 5 or higher (of a possible total 18) as indicative of symptoms of depression	Told by doctor they had high blood pressure, and/or those who were prescribed medications for HTN, and/or those whose SBP was 140 mmHg, and/or those whose DBP was 90 mmHg.	3‐years
(Zhao et al. 2023)	China	Cross‐Sectional	Middle‐Aged and Elderly Chinese Adults with Vascular Disease/Diabetes Mellitus	10,531	≥ 45	NA	The total CES‐D‐10 score ranges from 0 to 30, with a higher score indicating a higher level of depressive symptoms. A cut‐off score of ≥ 10was used for depression classification.	Interviews	NA

Abbreviation: BP, blood pressure; CIDI, Composite International Diagnostic Interview; CES‐D, Center for Epidemiologic Studies Depression Scale; CVD, Cardiovascular Disease; DBP, diastolic blood pressure, DSM, Diagnostic and Statistical Manual of Mental Disorders; DSM‐III, Diagnostic and Statistical Manual of Mental Disorders, Third Edition; DSM‐IV, Diagnostic and Statistical Manual of Mental Disorders, Fourth Edition; GDS‐CD, Geriatric Depression Scale‐Chinese Version; HADS, hospital anxiety and depression scale; HRQOL, health‐related quality of life; HTN, hypertension; ICD, International Classification of Diseases; IQR, Interquartile Range; JNC, Joint National Committee; NHANES, National Health and Nutrition Examination Survey; PAPI, Paper and Pencil Interviewing; PHQ, Patient Health Questionnaire; SBP, Systolic Blood Pressure; SF‐36 MH, Short Form‐36 Mental Health Subscale; WMH, World Mental Health.

The included studies presented a range of effect sizes for the association between various factors and the outcome of interest. The majority of studies reported OR, followed by HR and RR. Additionally, across research, lifestyle characteristics such as BMI, physical activity, alcohol use, and smoking were often controlled for. Comprehensive information is demonstrated in Table 2.

**TABLE 2:** Overview of association between depression and hypertension.

Study	Effect size	Adjusted RR/HR/OR (95%CI)	Adjusted variables
(Ackerman‐Banks et al. 2023)	OR	1.32 (1.17–1.50)	Maternal age at time of delivery, pre‐pregnancy depression, pre‐pregnancy HTN, pre‐pregnancy diabetes, obesity, smoking, nulliparity, pregnancy number in data set, year of delivery, Medicaid coverage during pregnancy, county‐level measures, zip code–level measures, hypertensive disorders of pregnancy, and gestational diabetes
(Amaike et al. 2024)	OR	7.75 (1.79–43.4)	Age, sex, physical exercise, cigarette smoking, and marital status
(Blümel et al. 2020)	OR	2.56 (1.77−3.70)	NA
(Cai et al. 2022)	OR	1.276 (1.114–1.462)	Age, gender, BMI, race, marital status, education, annual family income, alcohol drinking, smoking history, diabetes, stroke, and sleep duration
(Camacho et al. 2018)	OR	1.68 (1.09–2.60)	NA
(Choong et al. 2023)	OR	3.263 (2.053–5.18)	NA
(Duman et al. 2024)	OR	1.88 (1.10–3.21)	NA
(Fernald et al. 2021)	OR	Dutch = 1.53 (0.98–2.38) South‐Asian Surinamese = 1.10 (0.83–1.47) African Surinamese = 0.97 (0.71–1.33) Ghanaian = 1.00 (0.63–1.59) Turkish 1.17 (0.92–1.49) Moroccan = 0.88 (0.66–1.17)	Age, sex, and antidepressant drug use
(Flórez‐García et al. 2020)	OR	1.31 (1.09–1.57)	NA
(Gangwisch et al. 2010)	HR	1.31 (1.07–1.60)	Age, sex, race/ethnicity, education, body weight, diabetes, alcohol consumption, cigarette smoking, physical activity, pulse rate, sleep duration, and insomnia
(Ginty et al. 2013)	OR	1.19 (1.09–1.30)	Age, sex, socioeconomic status, smoking, sports participation, alcohol consumption, resting SBP, antidepressive and anxiolytic medication
(Grimsrud et al. 2009)	OR	Any depressive disorder = 1.37 (0.99–1.90) Major depressive disorder = 1.52 (1.07–2.17) Minor depressive disorder = 0.36 (0.11–1.18)	NA
(Han et al. 2008)	OR	1.67 (1.013–2.776)	NA
(Jackson et al. 2016)	OR	1.06 (0.95–1.18)	Age, anxiety, diabetes, heart disease & stroke, menopausal status, education and manage on income, marital status, all lifestyle factors, smoking, BMI, physical activity, alcohol status
(Jonas et al. 1997)	RR	2.99 (1.41–6.33)	NA
(Kabir et al. 2006)	OR	1.06 (1.01–1.10)	Sex, ethnicity, age group, predicted BMI, symptoms of depression, ethnicity
(Smith et al. 2024)	OR	1.00 (0.71–1.42)	Age, sex, marital status, living with others, heavy smoking, physical activity
(Liu et al. 2024)	OR	Moderate = 1.57(1.35–1.82) Severe = 1.99(1.34–2.96)	Age, gender, race, education, the ratio of family income to poverty, BMI, alcohol consumption status, smoking status, and diabetes
(Luo et al. 2023)	HR	1.24 (1.03–1.50)	Age, sex, dyslipidemia, diabetes, nephropathy, history of smoking, history of drinking
(Maatouk et al. 2016)	OR	1.67 (0.95–2.94)	Additional definition of HTN: Physician‐diagnosed HTN or self‐reported HTN and use of antihypertensive drugs, or HTN diagnosis at home visit
(Meyer et al. 2004)	OR	2.16 (0.94–4.98)	Age, race, gender, BMI, Nam‐powers score, alcohol use, smoking, exercise, diabetes status, and number of general medical visits
(Munezero and Tomita 2021)	OR	3.54 (1.10–11.37)	Age, marital status, household food insecurity, obesity, smoking status, alcohol consumption, physical activity, adverse childhood experiences (ACE), and depression severity
(Neyazi et al. 2024)	OR	1.014 (1.003–1.025)	NA
(Obas et al. 2022)	OR	1.86 (1.33–2.60)	Change in BP, and additionally the baseline systolic and diastolic BP, and the follow‐up duration of the wave
(Rhee et al. 2014)	OR	1.00 (0.87–1.13)	Age, marriage, cigarette smoking, alcohol use, exercise, education, cancer, angina, stroke, and thyroid disease
(Schuchman et al. 2024)	OR	Adults = 1.004 (0.982–1.025) Child = 1.012 (0.987–1.038)	Adjusted model for age, sex, race, BMI Z‐score, glomerular diagnosis, follow‐up time, edema, UPC, log eGFR, and steroid use
(Shah et al. 2023)	OR	2.72 (1.41–5.24)	Race/Hispanic origin, gender, and age
(Stein et al. 2010)	HR	Before 21 = 1.34 (1.20–1.48) After 21 = 1.15 (0.96–1.39)	Age, sex, country
(Tokioka et al. 2024)	OR	Male = 1.28 (0.65–2.5) Female = 1.29 (0.91–1.81)	BMI, research SBP, research HR, treated HT, HbA1c, LDL cholesterol, smoking status, alcohol intake, NaCl intake, regular exercise, use of sleeping pills, extent of house damage due to the Great East Japan Earthquake, educational status, season of the examination date, and year of the examination date
(Wang et al. 2021)	OR	2.83 (1.52–5.25)	Minimally sufficient adjustment set of variables retrieved from a literature‐based directed acyclic graphs (DAGs) and optimal adjustment set of variables derived from the least absolute shrinkage and selection operator (LASSO) regression
(Wen et al. 2010)	OR	1.434 (0.661–3.565)	Age, gender, BMI, satisfaction of sleep, smoking habits, alcoholic habits, tea habits, exercise habits, education levels, temperament
(Wiehe et al. 2006)	RR	1.15 (0.75–1.76)	Age, skin color, years at school, body mass index, beverage alcohol consumption, and physical activity at leisure time.
(Yan et al. 2003)	OR	1.17 (0.72–1.90)	Time urgency/impatience, achievement striving/competitiveness, hostility, depression, anxiety, age, black race, male sex, education, physical activity, Alcohol consumption, BMI, SBP
(Yousuf et al. 2022)	OR	0.998 (0.630–1.582)	NA
(Zambrana et al. 2016)	OR	1.53 (0.95–2.46)	Age, education, insurance, behavior, and clinical variables
(Zhao et al. 2023)	OR	3.17 (2.80–3.59)	Age, gender, education level, marital status, and health behaviors (i.e., drinking and smoking)

Abbreviations: ACE, adverse childhood experiences; BMI, body mass index; BP, blood pressure; CI, confidence interval; DAGs, directed acyclic graphs; eGFR, estimated glomerular filtration rate; HbA1c, haemoglobin A1c; HR, hazard ratio; LASSO, Least Absolute Shrinkage and Selection Operator; LDL, Low‐Density Lipoprotein; NA, not available; OR, odds ratio; RR, relative risk; SBP, systolic blood pressure; UPC, urinary protein‐to‐creatinine ratio.

### 3.3 Association Between Depression and Hypertension

**FIGURE 2:**
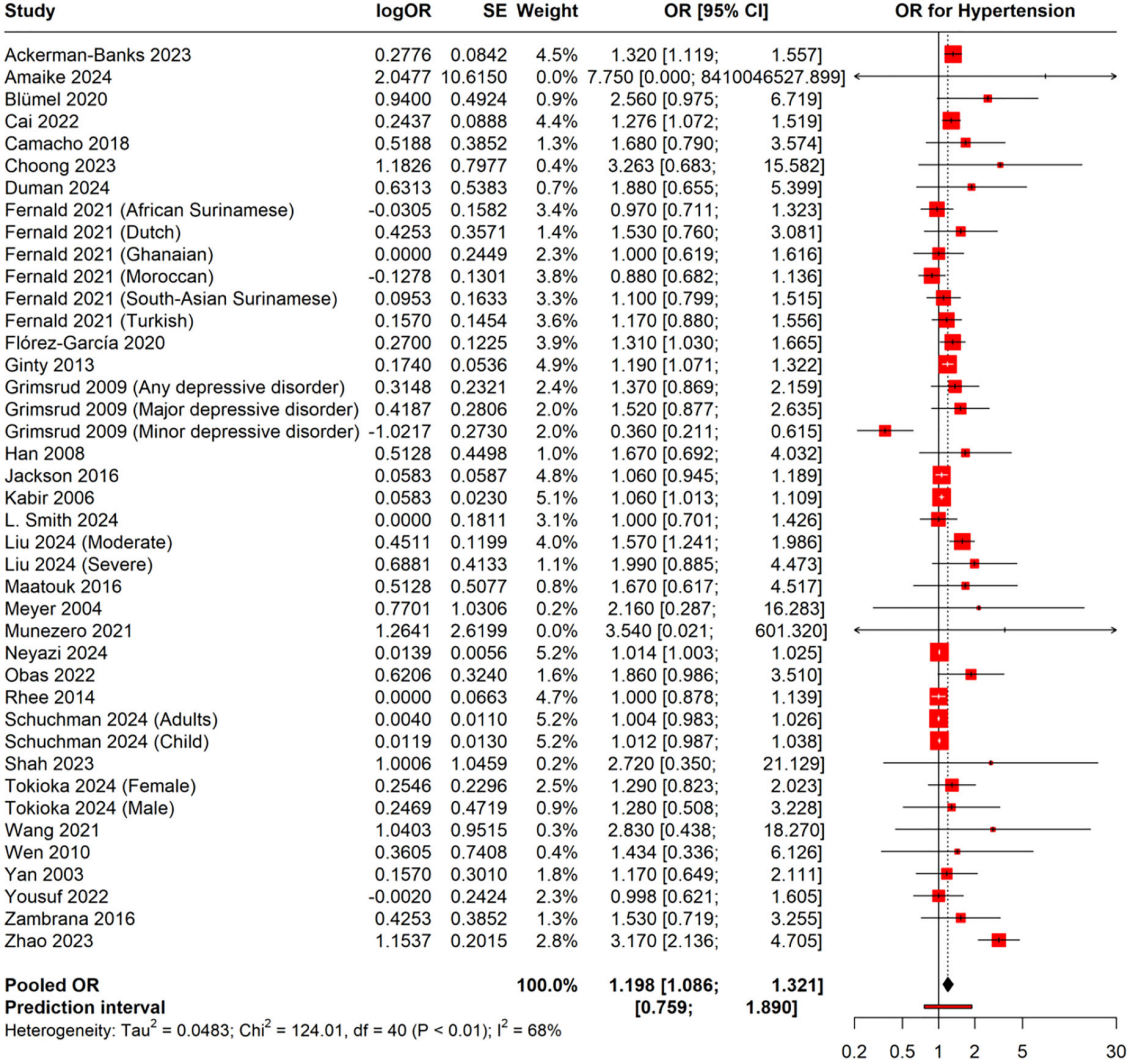
Forest plot showing OR of depression and risk of hypertension.

Depression and the risk of hypertension were shown to be significantly positively correlated in the pooled analysis (pooled OR = 1.198, 95% CI: 1.086–1.321). The analysis revealed substantial heterogeneity across studies (*I* ^2^ = 68%, *p* < 0.001, Tau^2^ = 0.0483), suggesting considerable variation in the observed associations (Figure 2).

**FIGURE 3:**
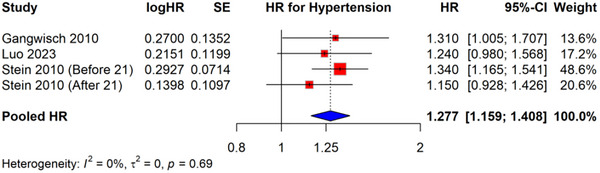
Forest plot showing HR of depression and risk of hypertension.

In the HR analysis of four longitudinal studies (Figure 3), depression was significantly associated with an increased odd of developing incident hypertension (pooled HR = 1.277, 95% CI: 1.159–1.408). Notably, there was no evidence of significant heterogeneity across studies (*I* ^2^ = 0%, *τ* ^2^ = 0, *p* = 0.69), suggesting consistency in the observed associations. All included studies demonstrated positive associations, with three of the four effect estimates showing statistical significance as indicated by CIs not crossing unity.

### 3.4 Subgroup and Sensitivity Analysis

**FIGURE 4:**
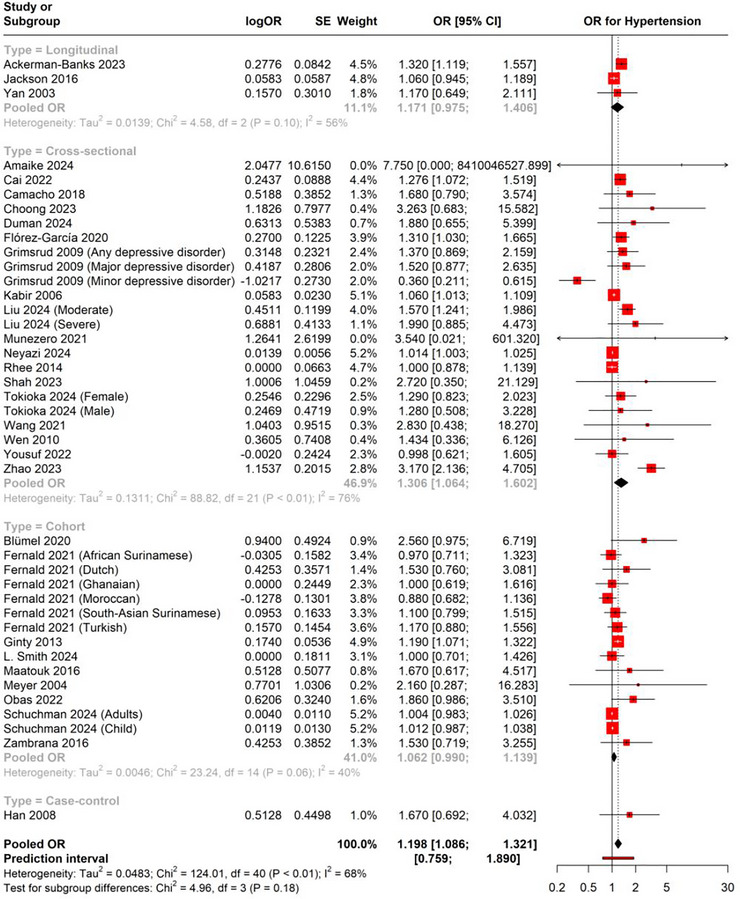
Subgroup analysis of OR.

**FIGURE 5:**
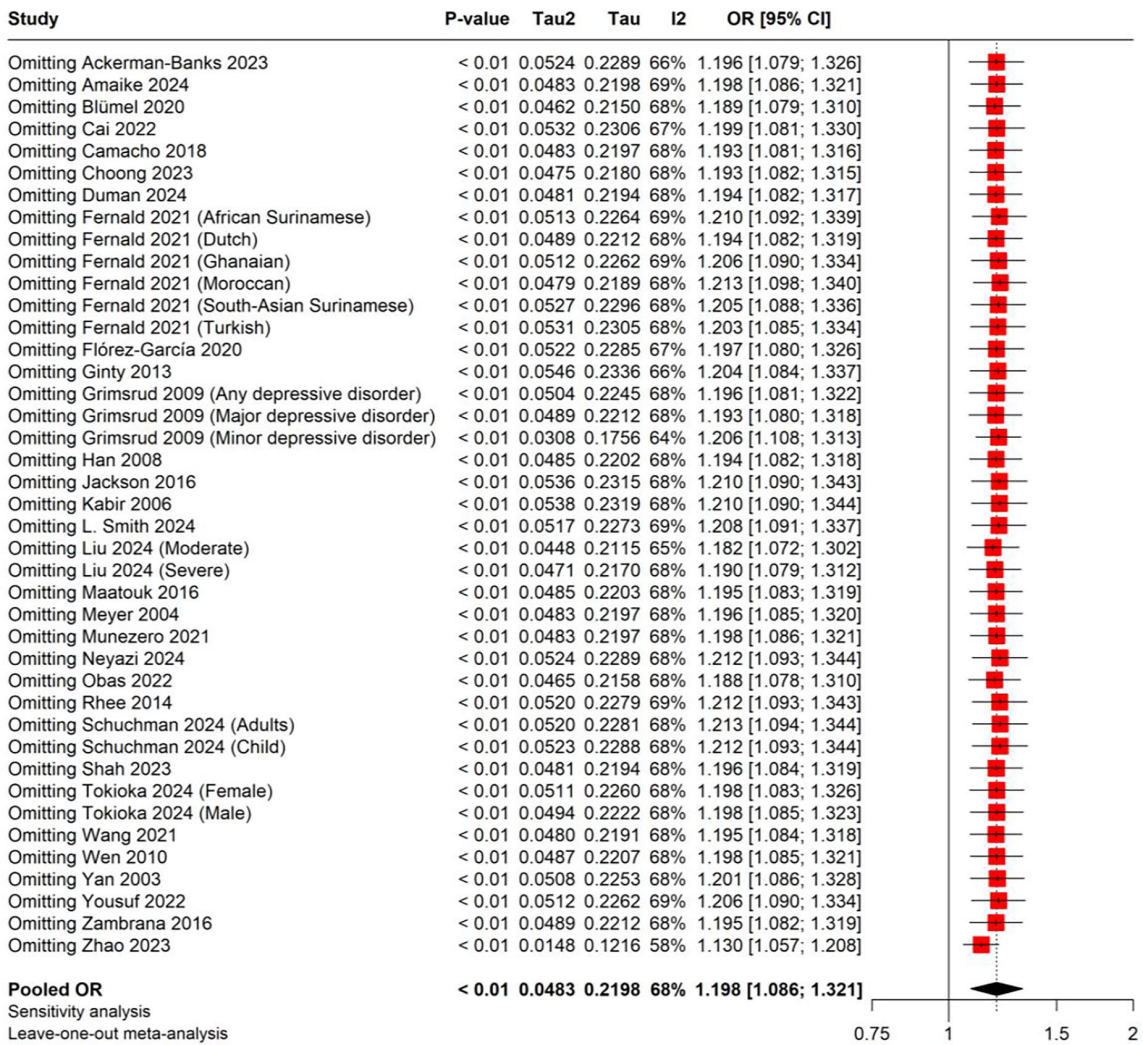
Sensitivity analysis of OR.

It was only in cross‐sectional studies with different levels of heterogeneity that this subgroup analysis revealed a positive correlation. In cross‐sectional studies, the pooled OR was 1.306 (95% CI: 1.064–1.602) with considerable heterogeneity (*I* ^2^ = 76%), whereas in longitudinal studies, the pooled OR was 1.171 (95% CI: 0.975–1.406) with moderate heterogeneity (*I* ^2^ = 56%). An OR of 1.062 (95% CI: 0.990–1.139) with heterogeneity (*I* ^2^ = 40%) was observed by cohort studies (Figure 4). By methodically eliminating one study at a time, the leave‐one‐out sensitivity analysis for OR evaluated the robustness of the pooled OR estimate. The findings showed that there was little change in the effect magnitude and that the pooled OR was constant across all rounds. There was a range of 1.130 (95% CI: 1.057–1.208) to 1.213 (95% CI: 1.094–1.344) for the pooled OR, confirming the robustness of the overall estimate. The heterogeneity throughout the analysis was *I* ^2^ = 68%, suggesting that 68% of the observed variance in effect sizes across studies is likely due to true differences between the studies rather than chance, indicating substantial heterogeneity and potentially influencing the reliability of the pooled estimate (Figure 5).

### 3.5 Publication Bias

**FIGURE 6:**
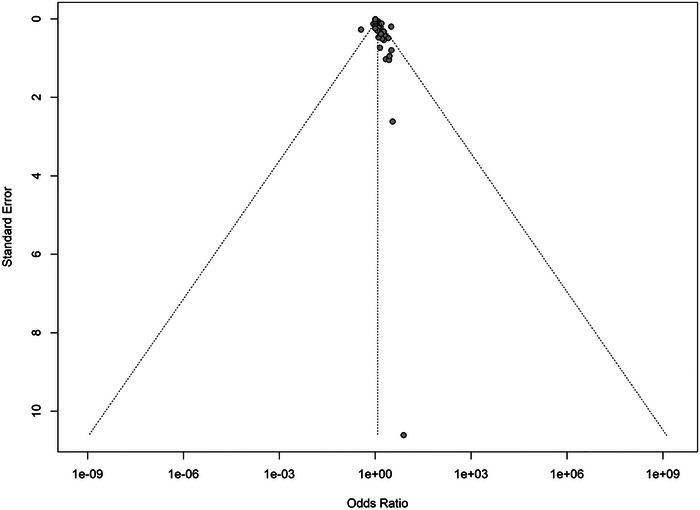
Funnel plot.

Asymmetry in the funnel plot raises the possibility of publishing bias. This is further supported by the statistically significant Egger's test (*p* = 0.0001), indicating that small studies with non‐significant results may be underrepresented in the literature. This publication bias may lead to an overestimation of the true effect size (Figure 6).

## 4 Discussion

This meta‐analysis and systematic review assessed the relationship between depression and the chance of hypertension. The results show that both illnesses are significantly positively correlated, with depression raising the risk of hypertension. This relationship persisted across various study designs, albeit with varying degrees of strength and heterogeneity. The implications of these findings indicate the need for a nuanced understanding of the interconnections between mental health and cardiovascular conditions to guide integrated prevention and treatment strategies.

Those with depression are more likely to have hypertension than people without depression, according to the analysis's pooled OR. The HR from longitudinal studies further supports the temporal relationship, suggesting that depression may precede the development of hypertension. These findings align with the hypothesis that depression contributes to cardiovascular dysregulation, potentially through mechanisms such as neuroendocrine disturbances, inflammation, and endothelial dysfunction. The absence of significant heterogeneity in HR from longitudinal studies (*I* ^2^ = 0%, *τ* ^2^ = 0, *p* = 0.69) contrasts with the substantial heterogeneity observed in cross‐sectional studies (*I* ^2^ = 76%). This suggests that longitudinal designs may offer more consistent estimates, possibly due to their ability to account for temporal sequences and control for confounding factors more effectively. The variability in cross‐sectional findings reflect the influence of study design, population characteristics, and methodological differences on observed associations. Subgroup analyses revealed significant variability based on study design and population characteristics. For instance, cross‐sectional studies demonstrated a stronger and significant association compared to cohort studies. This discrepancy may reflect differences in how depression and hypertension are assessed in these studies (Zou et al. 2024). While cohort studies usually utilize clinical diagnoses or longitudinal tracking, which provide more trustworthy data, cross‐sectional designs sometimes rely on self‐reported measures, which may be vulnerable to recall bias.

Several biological and behavioral mechanisms may explain the observed association between depression and hypertension. Chronic activation of the HPA axis in depression leads to sustained elevations in cortisol levels, which can induce vascular remodeling, increase arterial stiffness, and elevate blood pressure (Elsaid et al. 2021; Hui et al. 2024). Similarly, heightened sympathetic nervous system activity in depression may contribute to increased vascular resistance and hypertension (Lambert and Lambert 2011). Recent discoveries in mood disorders have identified a potential molecular link between bipolar disorder and hypertension, spurred by genome‐wide association studies implicating calcium channels in both conditions (Ferreira et al. 2008; Wan et al. 2024). In particular, it has been determined and confirmed that a locus encoding the gene CACNA1C, which codes for an L‐type calcium channel subunit, is a risk factor for bipolar illness (Craddock and Sklar 2013). Pathway analyses of genome‐wide association studies for bipolar illness have also found other calcium channel subunits, such as CACNAB2, a voltage‐gated calcium channel that may raise the risk of mood disorders and hypertension (Organization WH 2013; Luo et al. 2024). Compared to those without hypertension, Sandström and colleagues (Sandström et al. 2016) studied 298,414 individuals with a hypertension diagnosis and assessed the prevalence of depression, anxiety disorders, bipolar disorder, and schizophrenia. The main conclusion was that both men and women with hypertension had a somewhat higher chance of having a documented diagnosis of anxiety and depression than women did. Moreover, patients with anxiety disorders also have sympathetic predominance over lower parasympathetic activity, and higher BPV is linked to decreased baroreflex sensitivity. The start, progression, and degree of organ damage associated with hypertension are all predicted by elevated blood pressure (Zhou et al. 2021). The significance of the calcium/cAMP signaling pathway and intracellular calcium homeostasis disruption in the etiology of depression and hypertension is increasingly gaining attention (Bergantin 2020; Li et al. 2023). Calcium channel blockers have been demonstrated to alleviate a number of depression symptoms, including cognitive impairment, in addition to their antihypertensive effects. This may be due to a combination of the modulation of neurotransmitters like serotonin and sympathetic outflow (Bergantin 2020).

Poor diet in males but not in women, and physical inactivity in both sexes were substantially correlated with anxiety and sadness. In men, smoking behaviors were strongly connected with both anxiety and depression, whereas in women, smoking was only linked with depression. The global score, which represented unhealthy lifestyles in both sexes, was highly connected with the level of worry and despair (Bonnet et al. 2005; Shabil et al. 2023). A meta‐analysis of 36 prospective trials showed that increasing physical activity substantially reduced the future risk of incident depression during a mean follow‐up time of 7.4 years (OR = 0.837, 95% CI: 0.794–0.883), with minimal heterogeneity between included studies (*I* ^2^ = 0%) (Schuch et al. 2018). During a 1–6 year follow‐up, smokers had a substantially higher risk of developing depression than non‐smokers, according to a meta‐analysis of six studies including 15,333 teenagers aged 13–19 (OR = 1.73, 95% CI: 1.32–2.4) (Chaiton et al. 2009). Many individuals with depression exhibit behaviors that increase cardiovascular risk, such as sedentary lifestyles and unhealthy eating patterns. The role of SES is also noteworthy, as lower SES is associated with both higher prevalence of depression and limited access to healthcare, which can hinder hypertension prevention and management (Chaiton et al. 2009; Shabil et al. 2023).

The findings showed the importance of recognizing depression as a potential risk factor for hypertension. Integrating mental health screening into routine cardiovascular care could help identify individuals at higher risk of hypertension and facilitate early intervention. Clinicians should also consider the psychological impact of hypertension on patients, as the bidirectional relationship suggests that managing hypertension may reduce the likelihood of depressive symptoms. Considering the effects of antidepressants on blood pressure, the Blood pressure fluctuations in people with anxiety and depression disorders must be taken into account when using antidepressants. Antidepressant use is an independent predictor for the development of hypertension in men but not in women, according to the National Study of Adolescent to Adult Health (ADD Health), a longitudinal study that tracked adolescence into adulthood. The average diastolic blood pressure increase was 1.6 mmHg (Hamam et al. 2020; Bushi et al. 2023). Pharmacological interventions targeting shared pathways, such as anti‐inflammatory agents or beta‐blockers with mood‐stabilizing properties, may offer dual benefits in managing both conditions. Lifestyle interventions, including exercise, dietary modifications, and stress reduction techniques, should also be prioritized as they address both physical and mental health simultaneously.

There is substantial variation in the definitions and assessment methods employed across the included studies for both depression and hypertension, which may introduce measurement bias and affect the reliability of the pooled estimates. Depression was variably ascertained through clinical diagnoses based on DSM‐IV or ICD criteria in some studies, while others relied on self‐reported screening tools such as the CES‐D, HADS, or PHQ‐9, each with differing cut‐off scores (e.g., CES‐D ≥ 16 for probable depression). Similarly, hypertension definitions ranged from objective clinical measurements (e.g., systolic/diastolic blood pressure ≥ 140/90 mmHg or ≥ 130/80 mmHg) to self‐reported diagnoses or antihypertensive medication use, potentially leading to misclassification biases overestimation in self‐report reliant studies due to recall or social desirability biases, or underestimation in clinical measurement‐based studies if transient elevations were captured. These methodological inconsistencies likely contributed to the observed heterogeneity (*I* ^2^ = 68% overall, and up to 76% in cross‐sectional subgroups), inflating or attenuating effect sizes in certain designs and potentially biasing the pooled OR (= 1.198) toward a modest positive association. While subgroup analyses by study design partially mitigated this by revealing stronger associations in cross‐sectional studies (possibly due to greater reliance on self‐reports), future research should prioritize standardized diagnostic criteria (e.g., uniform use of validated tools with consistent thresholds) to enhance comparability and reduce bias, thereby strengthening conclusions on the depression‐hypertension link.

A further limitation of this meta‐analysis is the lack of consideration for White Coat Hypertension (WCH), a phenomenon where blood pressure readings are elevated in clinical settings due to anxiety or stress but remain normal outside such environments. WCH is particularly relevant in the context of depression, as heightened psychological stress or anxiety associated with depressive disorders may amplify stress‐induced blood pressure elevations during clinical visits, potentially leading to misclassification bias in studies relying on single clinical measurements or self‐reported hypertension diagnoses. None of the included studies explicitly accounted for WCH, which may have been misclassified as true hypertension, particularly in cross‐sectional studies with higher heterogeneity (*I* ^2^ = 76%). Future research should incorporate ambulatory or home blood pressure monitoring to differentiate WCH from sustained hypertension, ensuring more accurate diagnoses and reducing bias in assessing the depression‐hypertension relationship.

Notwithstanding this meta‐analysis's advantages, there are a few drawbacks to take into account. First, the significant heterogeneity seen in cross‐sectional research points to variations in demographic characteristics, measuring techniques, and study design. For instance, differences in the definitions and diagnostic criteria for depression and hypertension likely contributed to inconsistent findings. Some studies relied on self‐reported measures, which are prone to reporting biases, while others used clinical assessments or validated questionnaires, which are more robust but less universally applied. Second, residual confounding remains a concern. Although many studies adjusted for lifestyle factors and comorbidities, unmeasured confounders, such as genetic predispositions, access to healthcare, and cultural attitudes toward mental health, may influence the observed associations. Third, studies with non‐significant findings may be underrepresented due to publication bias, which might inflate the pooled effect size, as shown by the funnel plot asymmetry and Egger's test results. Fourth, the reliance on observational studies limits the ability to infer causality. While longitudinal studies provide stronger evidence for a temporal relationship, experimental studies are needed to establish definitive causal pathways. Additionally, most studies included in this meta‐analysis focused on adult populations, limiting the generalizability of findings to children or adolescents, where the interplay between mental health and cardiovascular risk may differ.

To address these limitations, future research should prioritize standardized methods for diagnosing depression and hypertension to improve comparability across studies. Prospective cohort studies with detailed assessments of confounding variables and longer follow‐up periods are needed to elucidate the temporal dynamics of the association. Experimental studies investigating the impact of specific interventions, such as antidepressants or anti‐inflammatory therapies, on blood pressure regulation could provide valuable insights into causal mechanisms. Moreover, investigating the role of genetic and epigenetic factors may uncover novel pathways linking depression and hypertension. Studies exploring the intersection of other psychiatric disorders, such as anxiety, and cardiovascular conditions could also enhance understanding of the broader mental‐physical health nexus. Finally, incorporating diverse populations, including children, adolescents, and individuals from underrepresented regions, will ensure that findings are generalizable across age groups and cultural contexts.

## 5 Conclusion

Depression and the risk of hypertension are significantly positively correlated, according to this comprehensive review and meta‐analysis. The findings emphasize the interconnected nature of mental and physical health and the need for integrated approaches to prevention and treatment. Addressing depression in individuals with or at risk for hypertension may not only improve mental health outcomes but also lessen the impact of heart disease. In order to enhance the general wellbeing of those impacted, future studies should try to unravel the intricate processes that underlie this connection and provide focused therapies.

## Author Contributions

Prakasini Satapathy conceived and designed the study, conducted the review, and drafted the manuscript. Abhay M. Gaidhane designed the methodology, analyzed data, and revised the manuscript; Nasir Vadia collected and analyzed data, and revised the manuscript; Soumya V Menon conducted the literature search and assisted with data extraction; Kattela Chennakesavulu supported data analysis and manuscript revision; Rajashree Panigrahi contributed to clinical implications and manuscript review; Ganesh Bushi assisted with data collection and manuscript editing; Mahendra Singh provided statistical expertise and reviewed the manuscript; Sanjit Sah contributed to data analysis and manuscript drafting; Khang Wen Goh helped with statistical analysis and writing; Muhammed Shabil coordinated the study and contributed to the final draft; Edward Mawejje supervised the study and reviewed the manuscript.

## Consent

The authors have nothing to report.

## Conflicts of Interest

The authors declare no conflicts of interest.

## Supporting information




**Supplementary Materials**: brb370931‐sup‐0001‐SuppMat.docx

## Data Availability

The data are available in the manuscript and associated supplementary file.
