# Correction to “Association between Depression and Risk of Hypertension: A Systematic Review and Meta‐Analysis”

**DOI:** 10.1002/brb3.71009

**Published:** 2025-10-24

Satapathy, P., A. M. Gaidhane, N. Vadia, et al. 2025. “Association Between Depression and Risk of Hypertension: A Systematic Review and Meta‐Analysis.” *Brain and Behavior* 15, no. 9: e70931. https://doi.org/10.1002/brb3.70931

In the originally published version, the title was incorrectly rendered as: Between Depression and Risk of Hypertension: A Systematic Review and Meta‐Analysis.

The correct title is: Association Between Depression and Risk of Hypertension: A Systematic Review and Meta‐Analysis.

The online version has been corrected accordingly.

We apologize for this error.

