# Supplementary material for: Association Between Depression and Risk of Hypertension: A Systematic Review and Meta‐Analysis
Source: Brain Behav. 2025 Sep 25;15(9):e70931. doi: 10.1002/brb3.70931 (PMC12463727; doi:10.1002/brb3.70931)
Supplement: Supplementary file 1 — Supplementary Materials: brb370931‐sup‐0001‐SuppMat.docx [file BRB3-15-e70931-s001.docx]

**Supplementary Materials**

**Table S1.** PRISMA Checklist

**Table S2.** The adjusted search terms as per searched electronic databases

**Table S3.** Newcastle-Ottawa Scale for the quality assessment of studies

## **Table S1.** PRISMA Checklist

| **Section and Topic** | **Item #** | **Checklist item** | **Location where item is reported** |
| --- | --- | --- | --- |
| **TITLE** | | |  |
| Title | 1 | Identify the report as a systematic review. | 1 |
| **ABSTRACT** | | |  |
| Abstract | 2 | See the PRISMA 2020 for Abstracts checklist. (made as per the Journal guidelines) | 2 |
| **INTRODUCTION** | | |  |
| Rationale | 3 | Describe the rationale for the review in the context of existing knowledge. | 3 |
| Objectives | 4 | Provide an explicit statement of the objective(s) or question(s) the review addresses. | 3 |
| **METHODS** | | |  |
| Eligibility criteria | 5 | Specify the inclusion and exclusion criteria for the review and how studies were grouped for the syntheses. | 4 |
| Information sources | 6 | Specify all databases, registers, websites, organisations, reference lists and other sources searched or consulted to identify studies. Specify the date when each source was last searched or consulted. | 4, Table S3 |
| Search strategy | 7 | Present the full search strategies for all databases, registers and websites, including any filters and limits used. | Table S3 |
| Selection process | 8 | Specify the methods used to decide whether a study met the inclusion criteria of the review, including how many reviewers screened each record and each report retrieved, whether they worked independently, and if applicable, details of automation tools used in the process. | 4 |
| Data collection process | 9 | Specify the methods used to collect data from reports, including how many reviewers collected data from each report, whether they worked independently, any processes for obtaining or confirming data from study investigators, and if applicable, details of automation tools used in the process. | 4 |
| Data items | 10a | List and define all outcomes for which data were sought. Specify whether all results that were compatible with each outcome domain in each study were sought (e.g., for all measures, time points, analyses), and if not, the methods used to decide which results to collect. | 3 |
|  | 10b | List and define all other variables for which data were sought (e.g., participant and intervention characteristics, funding sources). Describe any assumptions made about any missing or unclear information. | 4, Table 1 |
| Study risk of bias assessment | 11 | Specify the methods used to assess risk of bias in the included studies, including details of the tool(s) used, how many reviewers assessed each study and whether they worked independently, and if applicable, details of automation tools used in the process. | Table S3 |
| Effect measures | 12 | Specify for each outcome the effect measure(s) (e.g. risk ratio, mean difference) used in the synthesis or presentation of results. | 5 |
| Synthesis methods | 13a | Describe the processes used to decide which studies were eligible for each synthesis (e.g. tabulating the study intervention characteristics and comparing against the planned groups for each synthesis (item #5)). | 4,5 |
|  | 13b | Describe any methods required to prepare the data for presentation or synthesis, such as handling of missing summary statistics, or data conversions. | NA |
|  | 13c | Describe any methods used to tabulate or visually display results of individual studies and syntheses. |  |
|  | 13d | Describe any methods used to synthesize results and provide a rationale for the choice(s). If meta-analysis was performed, describe the model(s), method(s) to identify the presence and extent of statistical heterogeneity, and software package(s) used. | 4.5 |
|  | 13e | Describe any methods used to explore possible causes of heterogeneity among study results (e.g. subgroup analysis, meta-regression). | 5 |
|  | 13f | Describe any sensitivity analyses conducted to assess robustness of the synthesized results. | 6 |
| Reporting bias assessment | 14 | Describe any methods used to assess risk of bias due to missing results in a synthesis (arising from reporting biases). | 5 |
| Certainty assessment | 15 | Describe any methods used to assess certainty (or confidence) in the body of evidence for an outcome. | NA |
| **RESULTS** | | |  |
| Study selection | 16a | Describe the results of the search and selection process, from the number of records identified in the search to the number of studies included in the review, ideally using a flow diagram. | Table S2 |
|  | 16b | Cite studies that might appear to meet the inclusion criteria, but which were excluded, and explain why they were excluded. | NA |
| Study characteristics | 17 | Cite each included study and present its characteristics. | 4,5 Table 1 |
| Risk of bias in studies | 18 | Present assessments of risk of bias for each included study. | Table S3 |
| Results of individual studies | 19 | For all outcomes, present, for each study: (a) summary statistics for each group (where appropriate) and (b) an effect estimate and its precision (e.g. confidence/credible interval), ideally using structured tables or plots. | Table 1, Figure 2 |
| Results of syntheses | 20a | For each synthesis, briefly summarise the characteristics and risk of bias among contributing studies. | 4 |
|  | 20b | Present results of all statistical syntheses conducted. If meta-analysis was done, present for each the summary estimate and its precision (e.g. confidence/credible interval) and measures of statistical heterogeneity. If comparing groups, describe the direction of the effect. | 5,4 Figure 2 |
|  | 20c | Present results of all investigations of possible causes of heterogeneity among study results. | 5, Figure 3 |
|  | 20d | Present results of all sensitivity analyses conducted to assess the robustness of the synthesized results. | Figure S2 |
| Reporting biases | 21 | Present assessments of risk of bias due to missing results (arising from reporting biases) for each synthesis assessed. | Figure 4 |
| Certainty of evidence | 22 | Present assessments of certainty (or confidence) in the body of evidence for each outcome assessed. | NA |
| **DISCUSSION** | | |  |
| Discussion | 23a | Provide a general interpretation of the results in the context of other evidence. | 5,6, 7 |
|  | 23b | Discuss any limitations of the evidence included in the review. | 7 |
|  | 23c | Discuss any limitations of the review processes used. | 7 |
|  | 23d | Discuss implications of the results for practice, policy, and future research. | 7 |
| **OTHER INFORMATION** | | |  |
| Registration and protocol | 24a | Provide registration information for the review, including register name and registration number, or state that the review was not registered. | 3 |
|  | 24b | Indicate where the review protocol can be accessed, or state that a protocol was not prepared. | 3 |
|  | 24c | Describe and explain any amendments to information provided at registration or in the protocol. | NA |
| Support | 25 | Describe sources of financial or non-financial support for the review, and the role of the funders or sponsors in the review. | 8 |
| Competing interests | 26 | Declare any competing interests of review authors. | 7 |
| Availability of data, code and other materials | 27 | Report which of the following are publicly available and where they can be found: template data collection forms; data extracted from included studies; data used for all analyses; analytic code; any other materials used in the review. | Supplementary Materials |

**Table S2. The adjusted search terms as per searched electronic databases**

| Database | Search query | Results |
| --- | --- | --- |
| PubMed | (((hypertension[Title/Abstract]) OR (hypertension[MeSH Terms])) OR ("high blood pressure"[Title/Abstract])) AND ((depression[MeSH Terms]) OR (depression[Title/Abstract])) | 11,213 |
| EMBASE | (('hypertension':ti,ab OR 'high blood pressure':ti,ab) AND ('depression':ti,ab)) | 18,195 |
| WOS  advanced | (hypertension OR "high blood pressure") AND (depression) | 10,400 |

**Table S3.** Newcastle-Ottawa Scale for the quality assessment of studies

| **STUDY** | **SELECTION (max 4 points)** | | | | **COMPARABILITY (max 2 points)** | **OUTCOME (max 3 points)** | | | SCORE (out of 9 for cohort and 6 for cross-sectional) |
| --- | --- | --- | --- | --- | --- | --- | --- | --- | --- |
|  | Representativeness | Selection | Ascertainment | Demonstration of the outcome of interest was not present at start of study | Comparability the basis of the design or analysis | Assessment of outcome | Was follow-up long enough for outcomes to occur? | Adequacy of the follow-up |  |
| Ackerman-Banks 2023 (1) | 1 | 1 | 1 | 1 | 1 | 1 | NA | 1 | 7 |
| Amaike 2024 (2) | 1 | 1 | 1 | NA | 1 | 1 | NA | NA | 5 |
| Blümel 2020 (3) | 1 | 1 | 1 | 1 | 1 | 1 | 1 | 1 | 8 |
| Cai 2022 (4) | 1 | 1 | 1 | NA | 2 | 1 | NA | NA | 6 |
| Camacho 2018 (5) | 1 | 1 | 1 | NA | 2 | 1 | NA | NA | 6 |
| Choong 2023 (6) | 1 | 1 | 1 | NA | 1 | 1 | 0 | NA | 5 |
| Duman 2024 (7) | 1 | 1 | 1 | NA | 2 | 1 | NA | NA | 6 |
| Fernald 2021 (8) | 1 | 1 | 1 | 1 | 2 | 1 | NA | 1 | 8 |
| Flórez-García 2020 (9) | 1 | 1 | 1 | NA | 1 | 1 | NA | NA | 5 |
| Gangwisch 2010 (10) | 1 | 1 | 1 | 1 | 1 | 1 | 1 | 1 | 8 |
| Ginty 2013 (11) | 1 | 1 | 1 | 1 | 1 | 1 | 1 | 1 | 8 |
| Grimsrud 2009 (12) | 1 | 1 | 1 | NA | 1 | 1 | NA | NA | 5 |
| Han 2008 (13) | 1 | 1 | 1 | NA | 2 | 1 | NA | NA | 6 |
| Jackson 2016 (14) | 1 | 1 | 1 | 1 | 1 | 1 | 1 | 1 | 8 |
| Jonas 1997 (15) | 1 | 1 | 1 | 1 | 2 | 1 | 1 | 1 | 9 |
| Kabir 2006 (16) | 1 | 1 | 1 | NA | 1 | 1 | NA | NA | 5 |
| L. Smith 2024 (17) | 1 | 1 | 1 | 1 | 2 | 1 | 1 | 1 | 9 |
| Liu 2024 (18) | 1 | 1 | 1 | NA | 1 | 1 | NA | NA | 5 |
| Luo 2023 (19) | 1 | 1 | 1 | 1 | 1 | 1 | 1 | 1 | 8 |
| Maatouk 2016 (20) | 1 | 1 | 1 | 1 | 1 | 1 | 1 | 1 | 8 |
| Meyer 2004 (21) | 1 | 1 | 1 | 1 | 1 | 1 | 1 | 1 | 8 |
| Munezero 2021 (22) | 1 | 1 | 1 | NA | 1 | 1 | NA | NA | 5 |
| Neyazi, 2024 (23) | 1 | 1 | 1 | NA | 1 | 1 | NA | NA | 5 |
| Obas 2022 (24) | 1 | 1 | 1 | 1 | 1 | 1 | 1 | 1 | 8 |
| Rhee 2014 (25) | 1 | 1 | 1 | NA | 2 | 1 | NA | NA | 6 |
| Schuchman 2024 (26) | 1 | 1 | 1 | 1 | 2 | 1 | 1 | 1 | 9 |
| Shah 2023 (27) | 1 | 1 | 1 | NA | 1 | 1 | NA | NA | 5 |
| Stein 2010 (28) | 1 | 1 | 1 | NA | 1 | 1 | NA | NA | 5 |
| Tokioka 2024 (29) | 1 | 1 | 1 | NA | 1 | 1 | NA | NA | 5 |
| Wang 2021 (30) | 1 | 1 | 1 | NA | 2 | 1 | NA | NA | 6 |
| Wen 2010 (31) | 1 | 1 | 1 | NA | 2 | 1 | NA | NA | 6 |
| Wiehe 2006 (32) | 1 | 1 | 1 | NA | 2 | 1 | NA | NA | 6 |
| Yan 2003 (33) | 1 | 1 | 1 | 1 | 1 | 1 | 1 | 1 | 8 |
| Yousuf 2022 (34) | 1 | 1 | 1 | NA | 1 | 1 | NA | NA | 5 |
| Zambrana 2016 (35) | 1 | 1 | 1 | 1 | 1 | 1 | 1 | 1 | 8 |
| Zhao 2023 (36) | 1 | 1 | 1 | NA | 1 | 1 | NA | NA | 5 |

1. Ackerman-Banks CM, Lipkind HS, Palmsten K, Pfeiffer M, Gelsinger C, Ahrens KA. Association of Prenatal Depression With New Cardiovascular Disease Within 24 Months Postpartum. J Am Heart Assoc. 2023;12(9).

2. Amaike C, Salami OF, Bamidele OT, Ojo AM, Otaigbe I, Abiodun O, et al. Association of depression and anxiety with uncontrolled hypertension: A cross-sectional study in Southwest Nigeria. Indian J Psychiatry. 2024;66(2).

3. Blümel JE, Carrillo-Larco RM, Vallejo MS, Chedraui P. Multimorbidity in a cohort of middle-aged women: Risk factors and disease clustering. Maturitas. 2020;137.

4. Cai Y, Chen M, Zhai W, Wang C. Interaction between trouble sleeping and depression on hypertension in the NHANES 2005-2018. BMC Public Health. 2022;22(1).

5. Camacho J, Echeverría G, Barros J, Maiz A, Rigotti A. Depression and stress are highly associated with hypertension, diabetes, and cardiovascular disease in Chilean population. Atherosclerosis Supplements. 2018;32:74.

6. Choong SM, Lee PY, Rashid AA. Prevalence of undiagnosed depression among patients with hypertension: A cross-sectional study of Malaysian primary care perspective. Malays Fam Physician. 2023;18.

7. Duman H, Duman H, Puşuroğlu M, Yılmaz AS. Anxiety disorders and depression are associated with resistant hypertension. Adv Clin Exp Med. 2024;33(2).

8. Fernald F, Snijder M, van den Born B-J, Lok A, Peters R, Agyemang C. Depression and hypertension awareness, treatment, and control in a multiethnic population in the Netherlands: HELIUS study. Intern Emerg Med. 2021;16(7).

9. Flórez-García V, Rojas-Bernal LÁ, Bareño-Silva J. Depression and sleep disorders related to hypertension: A cross-sectional study in Medellín, Colombia. Rev Colomb Psiquiatr (Engl Ed).49(2).

10. Gangwisch JE, Malaspina D, Posner K, Babiss LA, Heymsfield SB, Turner JB, et al. Insomnia and sleep duration as mediators of the relationship between depression and hypertension incidence. Am J Hypertens. 2010;23(1).

11. Ginty AT, Carroll D, Roseboom TJ, Phillips AC, de Rooij SR. Depression and anxiety are associated with a diagnosis of hypertension 5 years later in a cohort of late middle-aged men and women. J Hum Hypertens. 2013;27(3).

12. Grimsrud A, Stein DJ, Seedat S, Williams D, Myer L. The association between hypertension and depression and anxiety disorders: results from a nationally-representative sample of South African adults. PLoS One. 2009;4(5).

13. Han J, Yin X-M, Xu F, Hong X, Liang Y-Q, Wang Z-Y. [A case-control study on depression and anxiety in hypertensive patients]. Zhonghua Liu Xing Bing Xue Za Zhi. 2008;29(2).

14. Jackson CA, Pathirana T, Gardiner PA. Depression, anxiety and risk of hypertension in mid-aged women: a prospective longitudinal study. J Hypertens. 2016;34(10).

15. Jonas BS, Franks P, Ingram DD. Are symptoms of anxiety and depression risk factors for hypertension? Longitudinal evidence from the National Health and Nutrition Examination Survey I Epidemiologic Follow-up Study. Arch Fam Med.6(1).

16. Kabir AA, Whelton PK, Khan MM, Gustat J, Chen W. Association of symptoms of depression and obesity with hypertension: the Bogalusa Heart Study. Am J Hypertens. 2006;19(6).

17. L. Smith M, Gelaye B, C. Tsai A, L. Gradus J. Mediation of the association between depression and coronary heart disease by metabolic syndrome components Meghan L. Smith a, *, Bizu Gelaye b, c, Alexander C. Tsai c, d, Jaimie L. Gradus a, e. Annals of Epidemiology. 2024;92(2024).

18. Liu C, Ye Z, Chen L, Wang H, Wu B, Li D, et al. Interaction eefcts between sleep-related disorders and depression on hypertension among adults: a cross-sectional study. Liu et al BMC Psychiatry. 2024;24.

19. Luo Q, Bao K, Gao W, Xiang Y, Li M, Zhang Y. Joint effects of depressive status and body mass index on the risk of incident hypertension in aging population: evidence from a nationwide population-based cohort study. BMC Psychiatry. 2023;23(1).

20. Maatouk I, Herzog W, Böhlen F, Quinzler R, Löwe B, Saum K-U, et al. Association of hypertension with depression and generalized anxiety symptoms in a large population-based sample of older adults. J Hypertens. 2016;34(9).

21. Meyer CM, Armenian HK, Eaton WW, Ford DE. Incident hypertension associated with depression in the Baltimore Epidemiologic Catchment area follow-up study. J Affect Disord. 2004;83(2-3).

22. Munezero T, Tomita A. Hypertension and Its Associated Mental Health Challenges Among Female African Refugees in Durban, South Africa. J Nerv Ment Dis. 2021;209(11).

23. Neyazi A, Mohammadi AQ, Neyazi M, Timilsina S, Padhi BK, Griffiths MD. Hypertension, depression, and health-related quality of life among hospitalized patients in Afghanistan. J Hum Hypertens. 2024;38(6):529-37.

24. Obas KA, Kwiatkowski M, Schaffner E, Lang UE, Stolz D, Eze IC, et al. Depression and cardiovascular disease are not linked by high blood pressure: findings from the SAPALDIA cohort. Sci Rep. 2022;12(1).

25. Rhee SJ, Kim EY, Kim SH, Lee HJ, Kim B, Ha K, et al. Subjective depressive symptoms and metabolic syndrome among the general population. Prog Neuropsychopharmacol Biol Psychiatry. 2014;54.

26. Schuchman M, M. Brady T, A. Glenn D, R. Tuttle K, Cara‑Fuentes G, V. Levy R, et al. Association of mental health‑related patient reported outcomes with blood pressure in adults and children with primary proteinuric glomerulopathies. Journal of Nephrology. 2024;37.

27. Shah RM, Doshi S, Shah S, Patel S, Li A, Diamond JA. Impacts of Anxiety and Depression on Clinical Hypertension in Low-Income US Adults. High Blood Press Cardiovasc Prev. 2023;30(4).

28. Stein DJ, Scott K, Haro Abad JM, Aguilar-Gaxiola S, Alonso J, Angermeyer M, et al. Early childhood adversity and later hypertension: data from the World Mental Health Survey. Ann Clin Psychiatry. 2010;22(1).

29. Tokioka S, Nakaya N, Nakaya K, Kogure M, Hatanaka R, Chiba I, et al. The association between depressive symptoms and masked hypertension in participants with normotension measured at research center. Hypertens Res. 2024;47(3).

30. Wang L, Li N, Heizhati M, Li M, Yang Z, Wang Z, Abudereyimu R. Association of Depression with Uncontrolled Hypertension in Primary Care Setting: A Cross-Sectional Study in Less-Developed Northwest China. Int J Hypertens. 2021;2021.

31. Wen Z, Bi-Rong D, Chang-Quan H, Zhen-Chan L, Yuan Z, Hong-Mei W, et al. Depression and hypertension among Chinese nonagenarians and centenarians. Int J Geriatr Psychiatry. 2010;25(6).

32. Wiehe M, Fuchs SC, Moreira LB, Moraes RS, Pereira GM, Gus M, Fuchs FD. Absence of association between depression and hypertension: results of a prospectively designed population-based study. J Hum Hypertens. 2006;20(6).

33. Yan LL, Liu K, Matthews KA, Daviglus ML, Ferguson TF, Kiefe CI. Psychosocial factors and risk of hypertension: the Coronary Artery Risk Development in Young Adults (CARDIA) study. Jama. 2003;290(16):2138-48.

34. Yousuf FS, Arif A, Bibi R, Almas A. Association of Depression and Anxiety With Hypertensive Crisis: A Cross-Sectional Study From a Hospital Setting in Karachi, Pakistan. Cureus. 2022;14(9).

35. Zambrana RE, López L, Dinwiddie GY, Ray RM, Eaton CB, Phillips LS, Wassertheil-Smoller S. Association of Baseline Depressive Symptoms with Prevalent and Incident Pre-Hypertension and Hypertension in Postmenopausal Hispanic Women: Results from the Women's Health Initiative. PLoS One. 2016;11(4).

36. Zhao Y, Zhang Y, Teopiz KM, Lui LMW, McIntyre RS, Cao B. Presence of Depression Is Associated with Functional Impairment in Middle-Aged and Elderly Chinese Adults with Vascular Disease/Diabetes Mellitus-A Cross-Sectional Study. Int J Environ Res Public Health. 2023;20(2).
